# Supplementary material for: Influence of Mixed Valence on the Formation of Oxygen Vacancy in Cerium Oxides
Source: Materials (Basel). 2019 Dec 5;12(24):4041. doi: 10.3390/ma12244041 (PMC6947290; doi:10.3390/ma12244041)
Supplement: Supplementary file 1 [file materials-12-04041-s001.docx]

Article

Influence of Mixed Valence on the Formation of Oxygen Vacancy in Cerium Oxides

Gege Zhou ^1,^ ^2^, Wentong Geng ^2^, Lu Sun ^1^, Xue Wang ^1^, Wei Xiao ^1^, Jianwei Wang ^1^ and Ligen Wang ^1,^*

^1^ Materials Computation Center, GRIMAT Engineering Institute Co. Ltd., General Research Institute for Nonferrous Metals, Beijing 100088, China; zgg8997@126.com (G.Z.);[sunlu@grinm.com](mailto:sunlu@grinm.com) (L.S.); [wangxue@grinm.com](javascript:void(0);) (X.W.); [xiaowei@grinm.com](mailto:xiaowei@grinm.com) (W.X.); [wangjianwei@grinm.com](javascript:void(0);) (J.W.); [lg_wang1@yahoo.com](javascript:void(0);) (L.W.)

^2^ School of Materials Science and Engineering, University of Science and Technology Beijing, Beijing 100083, China; [geng@mater.ustb.edu.cn](javascript:void(0);)

***** Correspondence: lg_wang1@yahoo.com; Tel.:+86–10–6066–2753

Received: 6 November 2019; Accepted: 25 November 2019; Published: date

The structural input files

**Table S1.** POSCAR for CeO_2_ (Figure 1a).

| **CeO2** |  |  |
| --- | --- | --- |
| 1.0 |  |  |
| 5.4809997749 | 0.0000000000 | 0.0000000000 |
| 0.0000000000 | 5.4809997749 | 0.0000000000 |
| 0.0000000000 | 0.0000000000 | 5.4809997749 |
| O | Ce |  |
| 8 | 4 |  |
| Direct |  |  |
| 0.250000000 | 0.250000000 | 0.250000000 |
| 0.750000000 | 0.750000000 | 0.250000000 |
| 0.750000000 | 0.250000000 | 0.750000000 |
| 0.250000000 | 0.750000000 | 0.750000000 |
| 0.250000000 | 0.250000000 | 0.750000000 |
| 0.750000000 | 0.750000000 | 0.750000000 |
| 0.750000000 | 0.250000000 | 0.250000000 |
| 0.250000000 | 0.750000000 | 0.250000000 |
| 0.000000000 | 0.000000000 | 0.000000000 |
| 0.000000000 | 0.500000000 | 0.500000000 |
| 0.500000000 | 0.000000000 | 0.500000000 |
| 0.500000000 | 0.500000000 | 0.000000000 |

**Table S2.** POSCAR for Ce_2_O_3_ (Figure 1b).

| **Ce2O3** |  |  |
| --- | --- | --- |
| 1.0 |  |  |
| 3.8900001049 | 0.0000000000 | 0.0000000000 |
| −1.9450000525 | 3.3688389116 | 0.0000000000 |
| 0.0000000000 | 0.0000000000 | 6.1799998283 |
| O | Ce |  |
| 3 | 2 |  |
| Direct |  |  |
| 0.000000000 | 0.000000000 | 0.000000000 |
| 0.333333356 | 0.666666713 | 0.647000027 |
| 0.666666630 | 0.333333321 | 0.352999973 |
| 0.333333356 | 0.666666713 | 0.246700005 |
| 0.666666630 | 0.333333321 | 0.753300033 |

**Table S3.** POSCAR for CeO_2_ -Ce_2_O_3_ (Figure 1c).

| **CeO2-Ce2O3** |  |  |
| --- | --- | --- |
| 1.0 |  |  |
| 3.8261500000 | 0.0000000000 | 0.0000000000 |
| −1.9130750000 | 3.3135430500 | 0.0000000000 |
| 0.0000000000 | 0.0000000000 | 15.546132000 |
| O | Ce |  |
| 9 | 5 |  |
| Direct |  |  |
| 0.333340000 | 0.666660000 | 0.050072000 |
| 0.000000000 | 0.000000000 | 0.150197300 |
| 0.666660000 | 0.333340000 | 0.250320000 |
| 0.333340000 | 0.666660000 | 0.350460000 |
| 0.000000000 | 0.000000000 | 0.496000000 |
| 0.666660000 | 0.333340000 | 0.649540000 |
| 0.333340000 | 0.666660000 | 0.749680000 |
| 0.000000000 | 0.000000000 | 0.849802700 |
| 0.666660000 | 0.333340000 | 0.949928000 |
| 0.000000000 | 0.000000000 | 0.000000000 |
| 0.333340000 | 0.666660000 | 0.200260000 |
| 0.666660000 | 0.333340000 | 0.400465000 |
| 0.333340000 | 0.666660000 | 0.591790000 |
| 0.666660000 | 0.333340000 | 0.792038000 |
